# A Comparative Study of Proteolytic Mechanisms during Leaf Senescence of Four Genotypes of Winter Oilseed Rape Highlighted Relevant Physiological and Molecular Traits for NRE Improvement

**DOI:** 10.3390/plants5010001

**Published:** 2015-12-22

**Authors:** Alexandra Girondé, Marine Poret, Philippe Etienne, Jacques Trouverie, Alain Bouchereau, Françoise Le Cahérec, Laurent Leport, Marie-Françoise Niogret, Jean-Christophe Avice

**Affiliations:** 1 UMR INRA-UCBN 950 Ecophysiologie Végétale, Agronomie & Nutritions N.C.S., Université de Caen Normandie, F-14032 Caen, France; alexandra.gironde@gmail.com (A.G.); marine.poret@unicaen.fr (M.P.); philippe.etienne@unicaen.fr (P.E.); jacques.trouverie@unicaen.fr (J.T.); 2 INRA, UMR 1349 Institut de Génétique, Environnement et Protection des Plantes, Agrocampus Ouest, Université de Rennes 1, F-35653 Le Rheu, France; alain.bouchereau@univ-rennes1.fr (A.B.); francoise.le-caherec@univ-rennes1.fr (F.L.C.); laurent.leport@univ-rennes1.fr (L.L.); marie-francoise.niogret@univ-rennes1.fr (M.-F.N.)

**Keywords:** *Brassica napus*, N remobilization, genotypic variability, proteolysis, acidic proteases

## Abstract

Winter oilseed rape is characterized by a low N use efficiency related to a weak leaf N remobilization efficiency (NRE) at vegetative stages. By investigating the natural genotypic variability of leaf NRE, our goal was to characterize the relevant physiological traits and the main protease classes associated with an efficient proteolysis and high leaf NRE in response to ample or restricted nitrate supply. The degradation rate of soluble proteins and D1 protein (a thylakoid-bound protein) were correlated to N remobilization, except for the genotype Samouraï which showed a low NRE despite high levels of proteolysis. Under restricted nitrate conditions, high levels of soluble protein degradation were associated with serine, cysteine and aspartic proteases at acidic pH. Low leaf NRE was related to a weak proteolysis of both soluble and thylakoid-bound proteins. The results obtained on the genotype Samouraï suggest that the timing between the onset of proteolysis and abscission could be a determinant. The specific involvement of acidic proteases suggests that autophagy and/or senescence-associated vacuoles are implicated in N remobilization under low N conditions. The data revealed that the rate of D1 degradation could be a relevant indicator of leaf NRE and might be used as a tool for plant breeding.

## 1. Introduction

Annually, 85–90 million tons of nitrogenous fertilizers are used worldwide, and this is expected to increase at least three-fold by 2050 [1,2]. Nitrogen (N) fertilizers in excess may lead to water and soil pollution due to leaching and as well as emissions of greenhouse effect gas (nitrous oxide) involved in global warming. Oilseed rape, which is the third most cultivated oleaginous crop worldwide, needs a high level of N fertilizers (160–250 kg·ha^−1^) to reach its expected seed yield [3]. Moreover, the average N use efficiency (NUE) of winter oilseed rape (10 kg DM·kg^−1^ N available) is lower than other crops, such as winter barley, wheat and oats (23 to 27 kg DM·kg^−1^ N available) [4]. Indeed, despite the high capacity of oilseed rape to absorb mineral N [5], only 50% of the N fertilizer is recovered in seeds at harvest [6]. Consequently, in order to improve the agro-environmental balance of oilseed rape, the optimization of NUE is becoming one of the main challenges in current breeding programs.

NUE is the result of the N uptake efficiency (NUpE) and the N utilization efficiency (NUtE). Investigations of four spring genotypes [7] and 10 winter genotypes [8] have highlighted that genotypic variations of NUE of oilseed rape at vegetative stages were mainly related to NUtE. In addition, even though a high N uptake is an obvious prerequisite for high NUE, an improvement in NUtE could lead to the optimization of the NUE with a similar or a lower amount of N uptake, which is expected in the context of lower N inputs. NUtE includes two components: the N remobilization efficiency (NRE) and the N assimilation efficiency (NAE). An enhanced NAE can improve the NUE of Brassicaceae, as demonstrated by mutants of oilseed rape and *Arabidopsis*-overexpressing enzymes of N metabolism (glutamine synthetase, alanine aminotransferase, *etc.*) [9]. On the other hand, at the vegetative stages, the leaves of oilseed rape may fall on the soil after abscission with a high residual N (up to 3.5% of N in leaf dry matter) [10], which could lead to a N loss of 100 kg N·ha^−1^·year^−1^ [11]. In addition, the removal of 50% of leaves during vegetative stages has led to a 30% decrease in the number of pod walls, despite a higher growth and N uptake [12]. Moreover, simulations carried out by modeling studies have suggested that a decrease of 1% in the amount of residual N in fallen leaves may lead to a 5%–10% increase in seed yield of winter oilseed rape [13]. These results demonstrate that the N remobilization from leaves during vegetative stages is crucial for growth and seed yield. Consequently, the characterization of cellular mechanisms associated with an efficient foliar N remobilization would help to identify traits for the selection of genotypes with reduced N loss, higher seed yield and lower N inputs, and therefore decrease the economic cost and risks of pollution by excessive N fertilization.

In leaf cells of oilseed rape, N is mainly stored as proteins (70% to 90% of reduced *N*-compounds [14]). During N remobilization, proteins are mainly degraded into peptides and amino acids by different classes of proteases. The resulting amino acids are exported from the source organs to the sink tissues by phloem vessels, after a conversion into their transportable form [15,16] by enzymes of N metabolism [17]. A study of the amount of amino acids in various subcellular compartments in oilseed rape (including cytosol, vacuole and plastids) did not show any amino acid accumulation during leaf N remobilization (*cv*. Lirajet [18]). In addition, no accumulation of amino acids was observed in senescent leaves of 11 genotypes of winter oilseed rape having different leaf NREs [8,19]. As the conversion and export of amino acids seems efficient at vegetative stages, proteolysis is regarded as being the limiting factor in leaf NRE [20]. Indeed, the degradation of soluble proteins has been highly correlated with N remobilization in senescent leaves of 10 winter oilseed rape varieties [8].

Chloroplasts can contain up to 70% of the proteins of the mesophyll cell [14,21], making the dismantlement of these organelles of special interest in the study of N remobilization during leaf senescence. Ribulose 1,5-bisphophate carboxylase-oxygenase (Rubisco) is localized in the stroma and can represent 50% of the soluble proteins in the mesophyll cell (*i.e.*, 20%–30% of the N in the mesophyll cell [22,23,24,25,26]). In addition to Rubisco, the thylakoid-bound proteins of light harvesting complex II (LHCII) of photosystem II (PSII) contain up to 20% of the N of the cell, which also makes their degradation crucial for N remobilization [27]. Thylakoid-bound proteins (such as the PSII proteins) are stabilized in the membrane by chlorophylls, which need to be degraded for proteolysis [23]. Despite the high N content of the PSII proteins, their catabolism is less studied than that of Rubisco.

The first step of proteolysis of chloroplastic proteins such as Rubisco is thought to be performed by chloroplastic proteases [28], although no involvement of serine proteases (SPs) has been evident in the degradation of chloroplastic proteins during senescence of oilseed rape leaves. However, SPs have been characterized during leaf senescence of other Brassicaceae (*B. oleracea* [29]) and in particular the Deg and Clp proteases in *Arabidopsis* [30,31,32]. Deg proteases are especially involved in the efficient degradation of the thylakoid-bound protein D1 of the PSII in response to high light conditions [33]. Several Deg proteases are also able to degrade various proteins of the LHCII *in vitro* [31,32,34,35]. In addition, the D1 protein is degraded by the filamentation temperature-sensitive H (FtsH) which is a member of the metalloprotease (MP) family [36]. Two FtsHs are accumulated during senescence of oilseed rape leaves in response to nitrate limitation or privation [19]. These proteases could also be crucial in the degradation of the lhcb1 and lhcb3 proteins of the LHCII in *Arabidopsis* [37], although a recent study contradicts this result [38]. In addition, an increase in metalloprotease activity is observed during post-harvest senescence of Broccoli [29] and a zinc-dependent metalloprotease of bean is able to degrade Rubisco *in vitro* [39]. An aspartic protease (AP) from tobacco, CND41 (chloroplast nucleoid DNA binding protein 41), is also believed to be involved in the Rubisco degradation at pH 7.5. In addition, a delayed senescence and a default in N remobilization were observed in a knock-out CND41 mutant, suggesting a crucial role of this AP for leaf proteolysis associated with senescence in tobacco [40,41]. A CND41 homologue was identified for *Arabidopsis* (56% identity) with a similar function [42], and a few proteins of *Brassica napus* have some similarities to CND41 (up to 52% identity), suggesting that a CND41 homologue exists in oilseed rape.

After the initial degradation in the chloroplasts by SP, AP and MP, further degradation by proteases from the vacuole and/or cytosol has been proposed, suggesting the involvement of subcellular trafficking. Indeed, proteins of the stroma (such as Rubisco and glutamine synthetase 2) were found in small vesicles (RCBs) [43], which are probably sent to the central lytic vacuole (linked to the mechanisms of autophagy) [44,45] and in small vacuoles (senescence-associated vacuoles; SAVs) where these proteins can be degraded by cysteine protease (CP; such as SAG12) and SP [28]. The fact that no PSII proteins were found in SAVs or RCBs [43,46] suggests that there are different pathways of degradation for stromal and thylakoid-bound proteins. Consequently, the proteolysis of thylakoid-bound proteins could be completely performed in chloroplasts during the first step of senescence while stromal proteins could be degraded via a pathway involving both chloroplast and extra-plastidic compartments [46]. However, CV-containing vesicles (CCVs), which are new vesicles formed at the final stage of chloroplast dismantling that have a potential vacuolar destination, have been recently shown to contain the thylakoid-bound protease FtsH1 [47]. In addition, these vesicles are associated with protein CV (chloroplast vesiculation), which has been linked to PSII destabilization, leading to a high susceptibility of the PSII thylakoid-bound proteins to chloroplastic proteases. Numerous vacuolar proteases of oilseed rape, such as CP and AP [19,48,49,50], have been proposed as being involved in the degradation of chloroplastic proteins during senescence in the lytic vacuole and SAVs [28]. More precisely, proteomic analyses have reported that the CP, SAG12, and an AP (GI: 1326165) are highly abundant during leaf senescence in response to nitrate limitation or privation [19] or present in dead leaves of oilseed rape *cv*. Capitol [51], suggesting a role for these specific proteases in the late phase of senescence. Finally, the β1 subunit of the 26S proteasome, mainly localized in the cytosol, has been shown to accumulate in senescent leaves of oilseed rape in response to a limitation/privation of nitrate [19], but its role remains unknown.

By investigating the natural genotypic variability of the leaf N remobilization observed in four genotypes of winter oilseed rape at vegetative stages (Aviso, Oase, Samouraï and Californium) [8], the present study aims to characterize the relevant physiological traits and the main protease classes associated with an efficient protein remobilization, which could be used as a tool for plant breeding. To reach this goal, the dynamics of leaf N remobilization were studied in relation to the changes in the amount of amino acids, the degradation of soluble proteins (including Rubisco) and thylakoid-bound proteins of the PSII (D1 and lhcb3), and the proteolytic activities at acidic and neutral pH.

## 2. Results

### 2.1. Changes in N and ^15^N Amounts during N Remobilization in the Source Leaf

**Figure 1:**
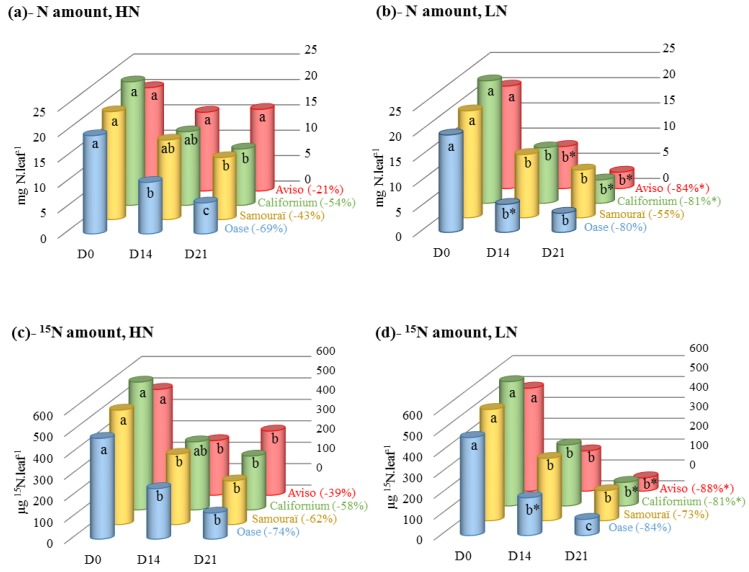
Changes in N and ^15^N amounts in the source leaves in response to restricted (LN, 0.375 mM) or ample (HN, 3.75 mM) nitrate applied for 21 days. The N and ^15^N amounts, estimated in HN (**a**,**c**) and LN (**b**,**d**) conditions, are expressed as mg N·leaf^−1^ and µg ^15^N·leaf^−1^, respectively. The percentage between brackets corresponds to the decrease in N and ^15^N at D21 as a percentage of the initial level (D0). D0: day 0; D14: day 14; D21: day 21. Data are expressed as the mean ± standard error (SE). For each genotype, the statistical differences in kinetics are indicated by letters a, b, c and a difference between N treatments is indicated by an asterisk (*n* = 3, *p* < 0.05).

N and ^15^N amounts were quantified to study the N remobilization in the source leaf in response to high (HN) or low (LN) nitrate regimes applied for 21 days. As expected, the N and ^15^N amounts throughout the experiment (after 14 days (D14) and 21 days (D21)) were correlated to the level of nitrate supply (Table 1) but an N supply × genotypes interaction was found solely for the N amount (*p* < 0.001). For all genotypes, both N and ^15^N amounts showed similar trends during the 21 days of experiment (Figure 1).

**Table 1:** Source of variation for the amount of N, ^15^N, soluble proteins and amino acids in the source leaves throughout the experiment. The plants were cultivated in restricted (LN, 0.375 mM) or ample (HN, 3.75 mM) nitrate supply. The main source of variation was deduced from an ANalysis Of VAriance (ANOVA) where N treatment (N), genotype (G), and N treatment × genotype (N×G) interactions were tested (*n* = 3, * *p* < 0.05; ** *p* < 0.01; *** *p* < 0.001). The resulting *F* values are also presented. The r values correspond to the correlation between the N supply and (i) the N; (ii) the ^15^N; (iii) the soluble protein; and (iv) the amino acid amounts *(n* = 12)*.*

N fractions	Source of Variation			
	N Treatment (N)		Genotype (G)	N × G
	*F*_N_	r	*F*_G_	*F*_NxG_
N amount	22.10 ***	0.45 **	8.39 ***	6.78 ***
^15^N amount	5.66 *	0.29 *	4.55 **	2.82
Soluble protein amount	5.95 *	0.33 *	1.28	1.14
Amino acid amount	44.62 ***	0.63 ***	5.31 **	3.74 *

Except for Aviso, all the genotypes presented a significant decrease in the N amount after 21 days of HN conditions (from −43% for Samouraï to −69% for Oase; Figure 1a). Compared with HN treatment, N remobilization was enhanced under LN conditions for Aviso (from D14) and Californium (at D21) to reach −84% and −81% at D21, respectively (Figure 1b). At D14, the leaf N amount in Oase was lower under LN conditions than in HN plants, but the final N remobilization was not significantly different to the HN treatment. Similarly, for Samouraï no difference in the N amount was observed between HN (−55%) and LN (−43%) conditions after 21 days of treatment. The ^15^N amount decreased for all genotypes in HN conditions (Figure 1c), with the greatest depletion observed in Oase (−74%) and the lowest in Aviso (−39%) at D21. In response to LN conditions (Figure 1d), an enhanced ^15^N remobilization was observed at D21 for Aviso (−88%) and Californium (−81%) alone. Contrastingly, Oase and Samouraï showed similar ^15^N remobilization at the endpoint (D21) under LN and HN conditions.

### 2.2. Changes in the Amounts of Soluble Proteins and Amino Acids during N Remobilization in Leaf

**Figure 2:**
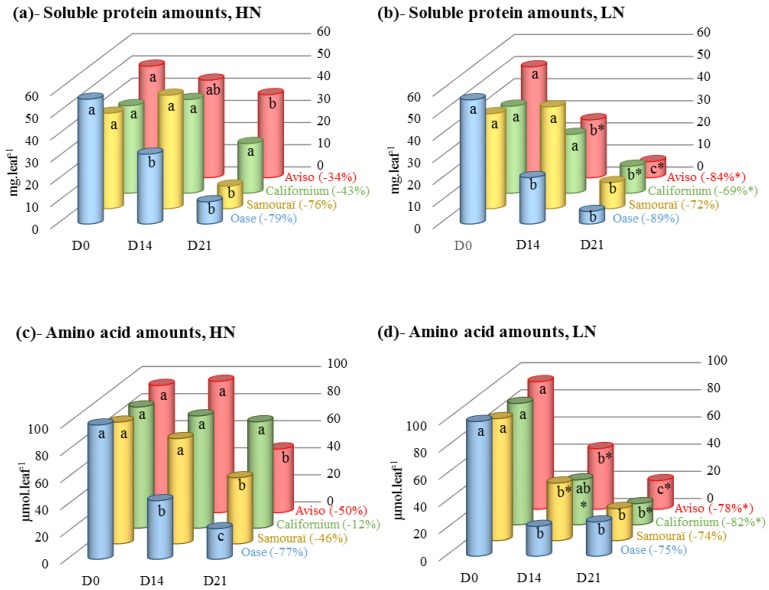
Changes in the amounts of soluble proteins and amino acids in the source leaves in response to restricted (LN, 0.375 mM) or ample (HN, 3.75 mM) nitrate applied for 21 days. The amounts of soluble proteins and ^15^N, estimated in HN (**a**,**c**) and LN (**b**,**d**) conditions, are expressed as mg·leaf^−1^ and µmol·leaf^−1^, respectively. The percentage between brackets corresponds to the remobilization of soluble proteins and amino acids at D21 as a percentage of the initial level (D0). D0: day 0; D14: day 14; D21: day 21. Data are expressed as the mean ± standard error (SE). For each genotype, the statistical differences in kinetics are indicated by letters a, b, c and a difference between N treatments is indicated by an asterisk (*n* = 3, *p* < 0.05).

The amount of soluble proteins in the source leaf depended on the N supply (*p* < 0.05), but no interaction between the N supply and the genotype was found (Table 1). Under HN conditions, the amount of soluble proteins decreased significantly for all genotypes except Aviso, with the greatest decline for Oase (−79% at D21; Figure 2a). Compared with HN conditions, a greater decrease in soluble proteins was observed in Aviso and Californium in response to LN treatment (−84% and −69% at D21, respectively; Figure 2b), while Oase and Samouraï had a similar decrease under LN conditions.

The amount of amino acids was related to N supply (*p* < 0.001) and genotype (*p* < 0.01) and an N supply × genotype interaction was found (*p* < 0.05; Table 1). Under HN conditions (Figure 2c), except for Californium, a significant decrease in the amino acid amount was observed for all genotypes (−77% for Oase, −50% for Aviso and −46% for Samouraï). For Aviso and Californium the decline in the amount of amino acids was larger in the LN than in the HN treatment after D14 and finally reached values of −78% and −82%, respectively, by D21 (Figure 2d). Compared with HN conditions, a lower amount of amino acids was observed at D14 for Samouraï under LN supply, but the final amount of amino acids was similar in both N treatments for Oase and Samouraï (Figure 2d).

### 2.3. Impact of Nitrate Supply on the Abundance of Rubisco Subunits and Thylakoid-Bound Proteins of Photosystem II (D1 and lhcb3)

**Figure 3:**
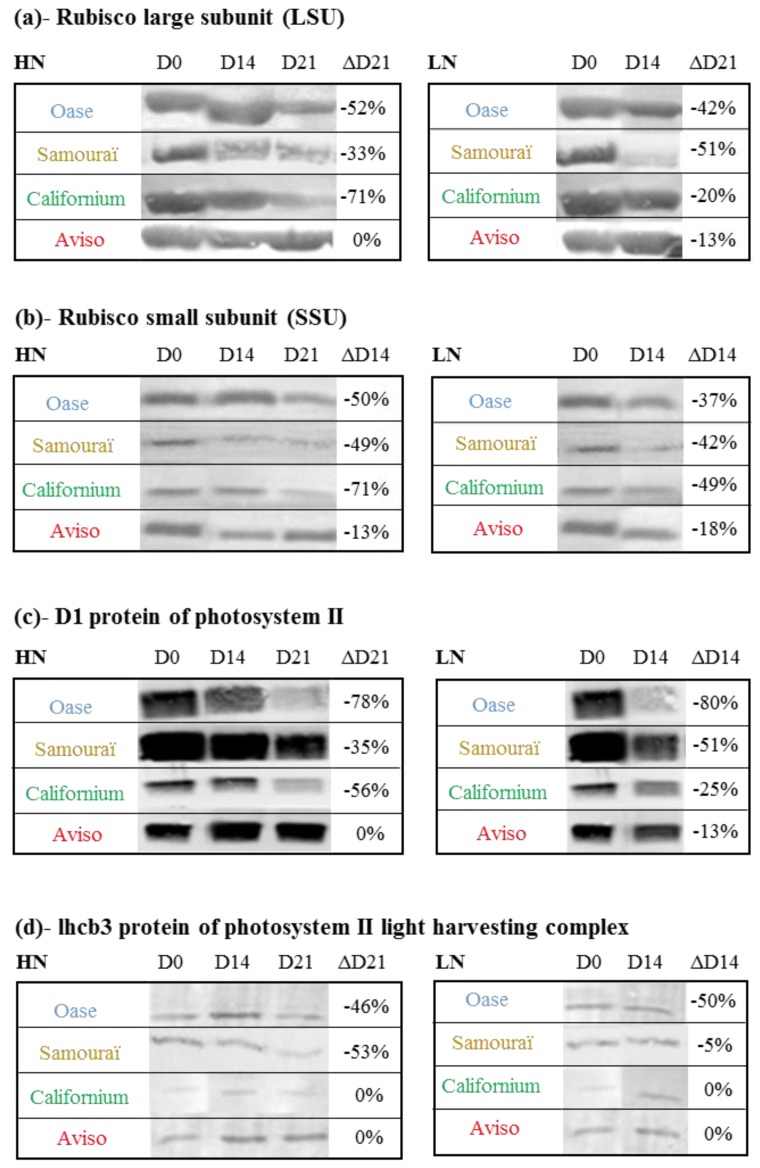
Immunodetection of large (LSU; (**a**)) and small (SSU; (**b**)) subunits of Rubisco, D1 protein of the photosystem II (**c**) and lhcb3 of the light harvesting complex (**d**) in the source leaves in response to restricted (LN, 0.375 mM) or ample (HN, 3.75 mM) nitrate applied for 21 days. The protein extracts of the three biological repetitions were pooled and 15 µg were loaded per lane for immunodetection. The protein abundance was determined with specific antibodies (see Experimental Section for details). D0: day 0; D14: day 14; D21: day 21. In LN conditions, immunodetection was not performed at D21 because there was not enough protein. The variation in protein abundance (ΔD21 or ΔD14) between D0 and D21 or D0 and D14 (when D21 is not available) is expressed as a percentage of the abundance observed at D0 and is given on the right of each immunoblot.

In order to determine if proteins of stroma (Large subunit (LSU) and Small subunit (SSU) of Rubisco) or thylakoid-bound proteins (D1 and lhcb3) are preferentially degraded in comparison to the rate of degradation observed for total soluble proteins during the senescence process, the changes of protein abundance were investigated by immunodetection after Western blotting (Figure 3). Under HN conditions, the abundance of Rubisco large subunit (LSU; Figure 3a) decreased by 71% for Californium, 52% for Oase and 33% for Samouraï, while no change in LSU abundance was observed for Aviso during the 21 days of treatment, meaning that this protein is not preferentially degraded in Aviso. The LSU abundance was similar in both N conditions for Californium and Aviso after 14 days, while a greater decrease of the LSU was observed at D14 under LN conditions for Oase and Samouraï. For the Rubisco small subunit (SSU; Figure 3b), the largest decrease under HN conditions was also observed for Californium (−71% at D21), followed by Oase and Samouraï (−50% at D21). The decline in SSU was similar between HN and LN treatments for Samouraï, Californium and Aviso, while a larger decrease in SSU abundance was observed in Oase at D14 in response to LN supply.

In response to HN conditions, the greatest decrease in D1 protein of photosystem II (Figure 3c) was observed for Oase (−78% at D21) and Californium (−56% at D21) while no change in D1 protein abundance was observed for Aviso during the 21 days of N treatment. In response to LN treatment, the decrease in D1 protein abundance occurred earlier for all genotypes and the largest decline was again observed for Oase (−80% at D14). Under HN conditions, the abundance of lhcb3 (Figure 3d), a protein that belongs to light harvesting complex II, declined substantially in the source leaf of Oase (−46%) and Samouraï (−53%) at D21, but not in Aviso and Californium. The abundance of lhcb3 was lower in LN than in HN Oase plants alone (−50% at D14).

### 2.4. Proteolytic Activities Related to Rubisco LSU Degradation and Identification of the Classes of Proteases Involved in Remobilization of the Source Leaf Proteins

**Figure 4:**
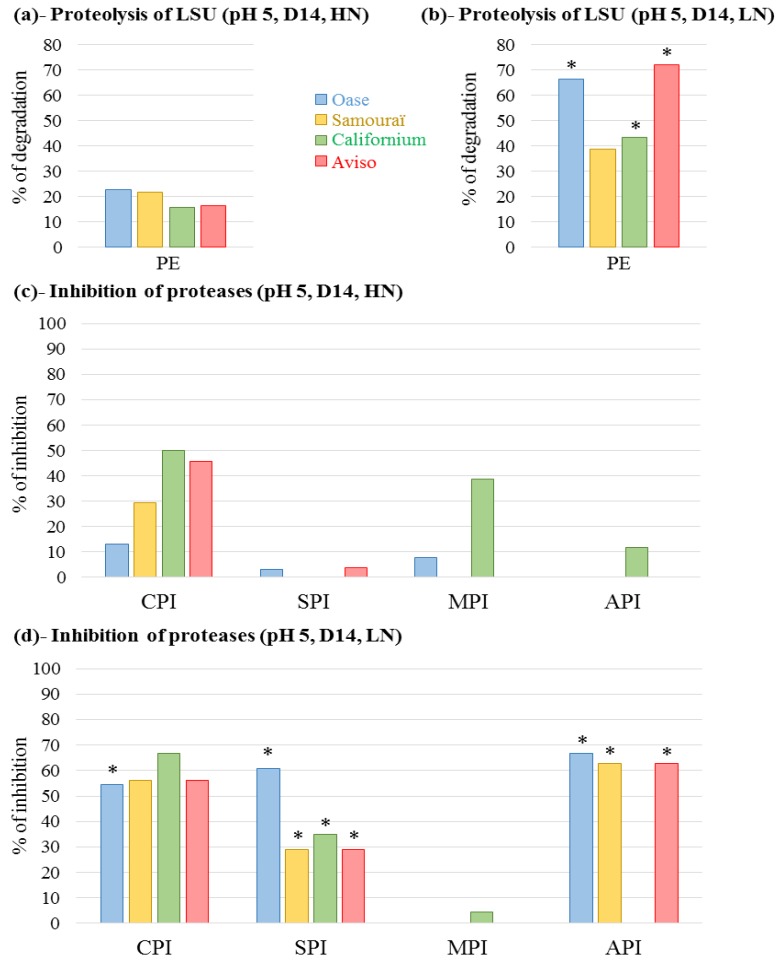
Total proteolytic activity at pH 5 at day 14 (D14; (**a**,**b**)) and the inhibition of cysteine proteases, serine proteases, metalloproteases and aspartic proteases by specific inhibitors (**c**,**d**). The plants were supplied with ample (HN, 3.75 mM; (**a**,**c**)) or restricted (LN, 0.375 mM; (**b**,**d**)) nitrate supply. The proteolytic activity corresponds to degradation of the Rubisco large subunit (LSU) visualized on Stain Free gels and quantified by Image Lab software (Bio-Rad) after incubation at 37 °C (for details see Experimental Section). The total proteolytic activity was determined with protein extract (PE) without protease inhibitor and is expressed as the percentage of degradation after 20 min of incubation. To determine the contribution of the different classes of proteases, the extract was incubated in the presence of specific protease inhibitors to determine the percentage of inhibition of LSU proteolysis observed without inhibitor (% inhibition; (**c**,**d**)). The inhibitors used were: iodoacetamide for cysteine proteases (CPI), aprotinin for serine proteases (SPI), 1–10 phenanthroline for metalloproteases (MPI) and pepstatin A for aspartic proteases (API). Due to the need to dissolve 1–10 phenanthroline and pepstatin A in methanol, the total proteolytic activity was also determined with methanol. The detailed gels are presented in Figure A1. For a given genotype, an asterisk is indicates if the percentage of degradation or the percentage of inhibition is at least two-fold increased in response to LN treatment compared with HN conditions.

**Figure A1:**
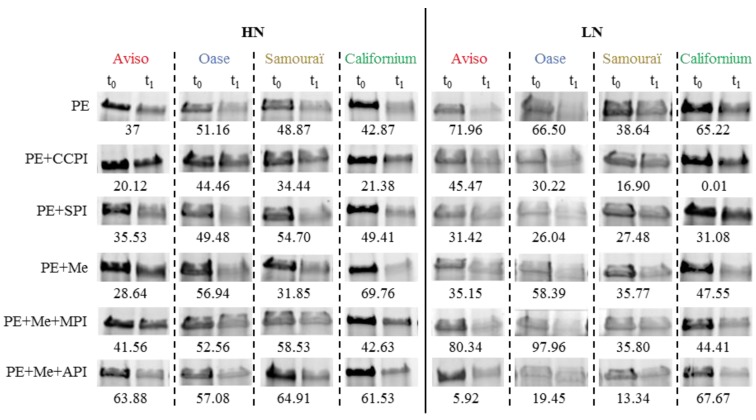
Total proteolytic activity at pH 5 (PE) and the inhibition of cysteine proteases (PE + CPI), serine proteases (PE + SPI), metalloproteases (PE + Me + MPI) and aspartic proteases (PE + Me + API) by specific inhibitors at day 14 (D14). The plants were supplied with ample (HN, 3.75 mM) or restricted (LN, 0.375 mM) nitrate supply. The proteolytic activity corresponds to degradation of the Rubisco large subunit (LSU) visualized on Stain Free gels and quantified by Image Lab software (Bio-Rad^®^) after incubation at 37 °C. The incubation was 20 min in LN conditions and 45 min in HN for all genotypes except Californium, which required 30 and 55 min, respectively. The percentage degradation is indicated below the gels. The inhibitors used were: iodoacetamide for cysteine proteases (CPI), aprotinin for serine proteases (SPI), 1–10 phenanthroline for metalloproteases (MPI) and pepstatin A for aspartic proteases (API). Due to the need to dissolve 1–10 phenanthroline and pepstatin A in methanol (Me), the total proteolytic activity was also determined with methanol (PE + Me).

Because a significant decrease in the soluble proteins was observed after 14 days under LN conditions (Figure 2b), the protease activities were first investigated at D14 through the development of an original method using the LSU of Rubisco as a substrate (see Experimental Section for details). Due to the large involvement of the vacuolar proteases in the degradation of the stromal proteins [52], the proteolytic activity was first characterized at pH 5 (Figure 4 and Figure A1).

Under HN conditions, Oase and Samouraï showed the highest proteolytic activity with a LSU degradation of 22.7% and 21.7%, while it was lower for Aviso (16.4%) and Californium (15.6%; Figure 4a). Under LN conditions, an increase in the proteolytic activities occurred for all genotypes and, contrary to HN conditions, Aviso had the greatest degradation (72%), followed by Oase (66.5%; Figure 4b). To define the classes of proteases involved in these proteolytic activities at pH 5, the LSU degradation was quantified in the presence of inhibitors of cysteine protease (CPI), serine protease (SPI), metalloprotease (MPI) and aspartate protease (API). Under HN conditions, CPI caused the largest inhibition of proteolysis for all genotypes: Californium (50.1%), Aviso (45.6%), Samouraï (29.5%) and Oase (13.1%) (Figure 4c). In response to LN conditions, the proteolysis of LSU was strongly inhibited by CPI for all genotypes (from 55.5% inhibition for Oase to 66.6% for Californium, Figure 4d). In addition, a low inhibition of LSU proteolysis was observed with the SPI for Aviso and Oase (4% and 3.3% inhibition, respectively) under HN conditions. Under LN treatment, the degradation of LSU was greatly inhibited by SPI for all genotypes, from 28.9% to 60.8% inhibition for Samouraï and Oase, respectively (Figure 4d). An inhibition of LSU proteolysis was observed in the presence of the MPI for Californium in both N conditions (38.9% and 4.4% of inhibition under HN and LN conditions, respectively; Figure 4c,d) and Oase under HN conditions (7.7% of inhibition; Figure 4c). The degradation of LSU was inhibited by the API solely for Californium under HN conditions (11.8%; Figure 4c). Under LN treatment, a substantial inhibition of LSU proteolysis by API was observed for all genotypes, except Californium (from 66.7% for Oase to 62.7% for Samouraï and Aviso) (Figure 4d).

**Figure 5:**
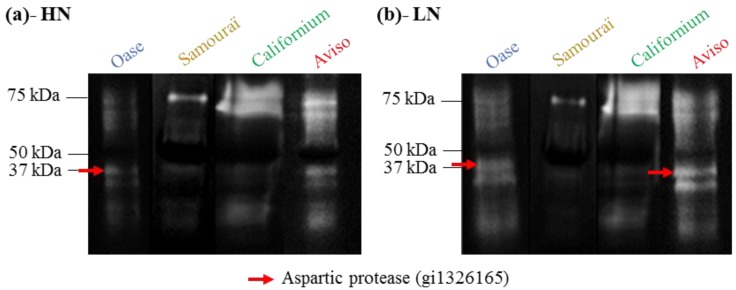
Zymograms of the proteolytic activities observed at pH 5 in the source leaves in response to ample (HN, 3.75 mM; (**a**)) or restricted (LN, 0.375 mM; (**b**)) nitrate applied for 14 days. The soluble proteins of the three biological repetitions at day 14 (D14) were pooled and 75 µg were loaded per lane. The white bands representing proteolytic activities at pH5 were analyzed to identify the proteases responsible under HN (**a**) and LN (**b**) conditions. One aspartic protease was successfully identified and is indicated by the red arrows (see Table A1 for details).

Zymograms were also performed at pH 5 to identify the vacuolar proteases whose activity was specifically induced in the leaf during N remobilization after 14 days of the experiment (Figure 5).

In both N conditions, proteolytic activities were detected at 70–75 kDa for Samouraï, Californium and Aviso, but the proteomic analysis of the corresponding bands by LC-MS/MS did not allow identification of a protease. Proteolytic activity was also found at 37 kDa for all genotypes, especially for Aviso and Oase under HN conditions (Figure 5a). In response to LN treatment, this 37 kDa proteolytic activity was enhanced compared with HN conditions for Aviso and Oase (Figure 5b). The proteomics analysis by LC-MS/MS showed that these activities under LN conditions were associated with an aspartic protease of *Brassica napus* (GI: 1326165; Table A1), previously found in senescing and dead leaves of winter oilseed rape *cv.* Capitol [19,51]. In addition, another proteolytic activity was found around 30 kDa for Oase and Aviso, and it was higher under LN (Figure 5b) than under HN conditions (Figure 5a), but the proteases were not identified by proteomics.

**Figure 6:**
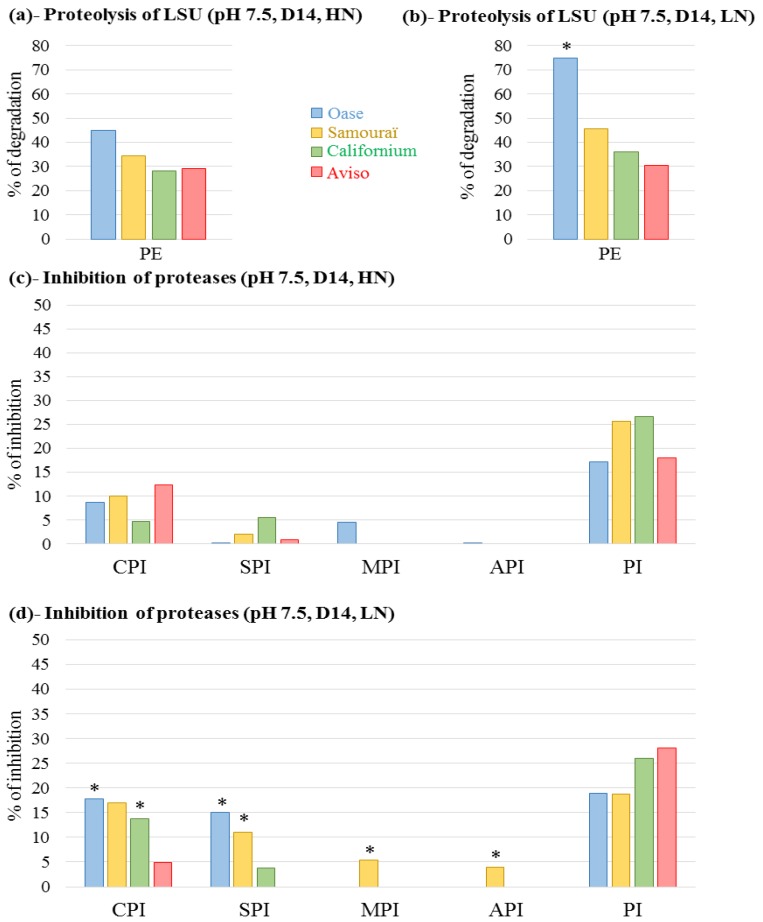
Total proteolytic activity at pH 7.5 at day 14 (D14; (**a**,**b**) and the inhibition of cysteine proteases, serine proteases, metalloproteases, aspartic proteases and proteasome by specific inhibitors (**c**,**d**)). The plants were supplied with ample (HN, 3.75 mM; (**a**,**c**)) or restricted (LN, 0.375 mM; (**b**,**d**)) nitrate supply. The proteolytic activity corresponds to the Rubisco large subunit (LSU) degradation visualized on Stain Free gels and quantified by Image Lab software (Bio-Rad) after an incubation at 37 °C. The total proteolytic activity was determined with protein extract (PE) without protease inhibitor and is expressed as the percentage of degradation after 60 min of incubation. To determine the contribution of the different classes of proteases, the extract was incubated in the presence of specific protease inhibitors to determine the percentage inhibition of LSU proteolysis observed without inhibitor (% inhibition; (**c**,**d**)). The inhibitors used were: iodoacetamide for cysteine proteases (CPIs), aprotinin for serine proteases (SPIs), 1–10 phenanthroline for metalloproteases (MPIs), pepstatin A for aspartic proteases (APIs) and MG132 (carbobenzoxy-Leu-Leu-leucinal) for proteasome (PI). Due to the need to dissolve 1–10 phenanthroline and pepstatin A in methanol and MG132 in DMSO, the total proteolytic activity was also determined with methanol and DMSO. The detailed gels are presented in Figure A2. For a given genotype, an asterisk is indicates if the % of degradation or the % of inhibition is increased at least two-fold in response to LN treatment compared with HN conditions.

**Figure A2:**
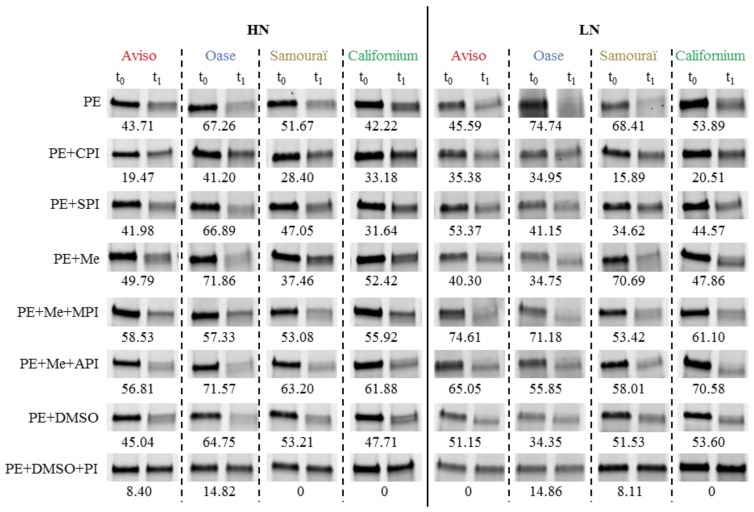
Total proteolytic activity at pH 7.5 (PE) and the inhibition of cysteine proteases (PE + CPI), serine proteases (PE + SPI), metalloproteases (PE + Me + MPI), aspartic proteases (PE + Me + API) and proteasome (PE + DMSO + PI) by specific inhibitors at day (D14). The plants were supplied with ample (HN, 3.75 mM) or restricted (LN, 0.375 mM) nitrate supply. The proteolytic activity corresponds to the Rubisco large subunit (LSU) degradation visualized on Stain Free gels and quantified by Image Lab software (Bio-Rad^®^) after an incubation at 37 °C. The incubation was 90 min in both N conditions for all genotypes except Oase, which was incubated for only 60 min. The percentage of degradation is indicated below the gels. The inhibitors used were: iodoacetamide for cysteine proteases (CPI), aprotinin for serine proteases (SPI), 1–10 phenanthroline for metalloproteases (MPI), pepstatin A for aspartic proteases (API) and MG132 (carbobenzoxy-Leu-Leu-leucinal) for proteasome (PI). Due to the need to dissolve 1–10 phenanthroline and pepstatin A in methanol (Me) and MG132 in DMSO, the total proteolytic activity was also determined with methanol (PE + Me) and DMSO.

Chloroplastic and cytosolic proteases are also thought to play an important role in N remobilization [52]. Consequently, the proteolytic activities were also studied at pH 7.5 (Figure 6 and Figure A2).

In both N conditions, the greatest total proteolytic activity was found for Oase (44.8% and 74.7% of LSU degradation under HN and LN conditions, respectively; Figure 6a,b) while the lowest activities were observed for Californium (28.2% and 35.9% under HN and LN conditions, respectively) and Aviso (30% in both N conditions). CPI inhibited the degradation of LSU in all genotypes under HN conditions, with the greatest inhibition in Aviso (12.3%) and the lowest in Californium (4.8%; Figure 6c). Under LN conditions, as at pH5, the inhibition of LSU degradation by CPI increased for Oase (17.7%), Californium (17%) and Samouraï (13.8%), while it decreased for Aviso (5%; Figure 6d). Under HN conditions, the proteolysis of LSU was weakly inhibited by SPI in the source leaf of Samouraï and Aviso, but a larger inhibition was observed for Californium (5.7%; Figure 6c). In response to LN supply, the degradation of LSU was more strongly inhibited by SPI for Oase (15%) and Samouraï (11%), whereas it decreased for Californium (3.8% of inhibition) and was not detectable for Aviso (Figure 6d). The inhibition of LSU degradation by MPI was only observed under HN conditions for Oase (4.5%; Figure 6c) and under LN conditions for Samouraï (5.4%; Figure 6d). Apart from Samouraï under LN conditions (4%; Figure 6d), no inhibition of LSU degradation by API was observed for the remaining genotypes under either of the N conditions. Finally, the greatest inhibition of LSU degradation was found in the presence of the proteasome inhibitor (PI), irrespective of the genotype or the N supply (Figure 6c,d).

**Figure 7:**
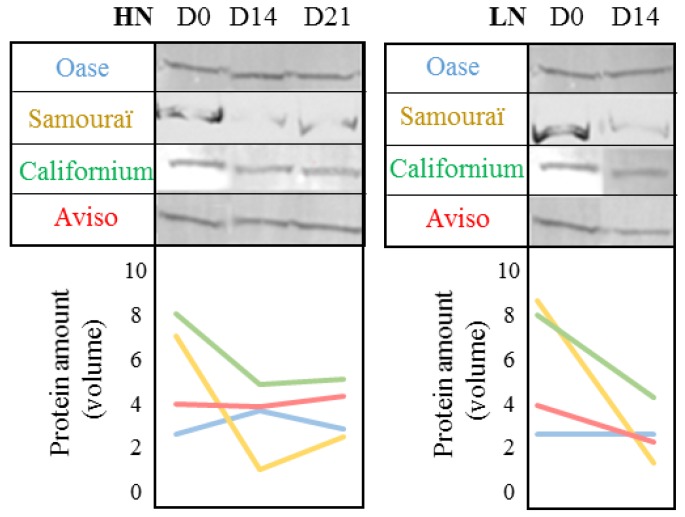
Abundance of FtsH proteases in the source leaf in response to ample (HN, 3.75 mM) or restricted (LN, 0.375 mM) nitrate applied for 21 days. The protein extracts of the three biological repetitions were pooled and 15 µg were loaded per lane for the detection of FtsH. The protein abundance was determined with specific antibodies (see Experimental Section for details). D0: day 0; D14: day 14; D21: day 21. In LN conditions, immunodetection was not performed at D21 because there was not enough protein. The graph below immunoblots represents the changes of protein abundance.

To better understand the involvement of the FtsH proteases (metalloproteases located in chloroplasts), which have been reported as having a role in leaf N remobilization [19,20], the abundance of these proteases during the 21 days of the experiment was also analyzed by immunodetection after Western blotting (Figure 7). In both N conditions (Figure 7), the amount of FtsH remained stable for Aviso and Oase or declined slightly for Californium during the 21 days. Contrastingly, in the source leaf of Samouraï, the abundance of FtsHs strongly decreased in both N treatments between D0 and D14 and became nearly undetectable compared to other genotypes.

## 3. Discussion

A recent exploration of the genotypic variability of NUE components of winter oilseed rape at vegetative stages revealed that a high biomass production under restricted N supply is linked to an enhanced NRE in leaves, and this is associated with a high N utilization efficiency of the N redistributed to growing leaves [8]. This previous work also confirmed that the amino acid export is generally efficient and the mechanisms of proteolysis seemed to be the main limiting factor for leaf NRE during sequential senescence. Consequently, the identification of proteolysis mechanisms associated with a high NRE would help to find new traits to select genotypes with higher biomass production under restricted N supply. To reach this goal, the mechanisms of proteolysis associated with N remobilization were studied in detail in a senescing leaves of four genotypes (Aviso, Oase, Samouraï and Californium) representing four different profiles of response to a limitation of nitrate supply, as previously reported by Girondé *et al.* [8].

### 3.1. The Improvement of Leaf NRE in Aviso and Californium in Response to LN Supply is Associated with a Higher Contribution of Acidic Proteases

For Aviso and Californium, N remobilization under HN conditions (Figure 1a) is limited by the degradation of soluble proteins (Figure 2a) and, especially for Aviso, by the degradation of the proteins D1 and lhcb3 (Figure 3c,d). In addition, the low N remobilization of the genotype Californium under HN conditions (Figure 1a) could be at least partially related to a low amino acid export (Figure 2c), suggesting that amino acids can partially limit the N remobilization. For both genotypes, the massive increase in N remobilization in response to LN supply (Figure 1b) is associated with (i) a strong degradation of soluble proteins (Figure 2b); (ii) an increase in amino acid export (Figure 2d); and, to a lesser extent, (iii) a higher degradation of the membrane protein D1 (Figure 3c). Taken together, these results highlight that an efficient proteolysis of soluble and thylakoid-bound proteins, especially the D1 proteins, is needed to reach a high leaf NRE.

For both genotypes, the increase in proteolysis under LN conditions is linked to an increase in proteolytic activity at pH 5 (Figure 4b) and this is associated with a large involvement of cysteine and serine proteases (Figure 4d). Due to the fact that no clear evidence for degradation of thylakoid-bound proteins by vacuolar proteases has been found [28], the high proteolytic activity at pH 5 under LN conditions is probably related to the high degradation of the soluble proteins. This result suggests that the N limitation induced subcellular trafficking of the chloroplastic proteins to the lytic vacuoles. This trafficking could imply the mechanisms of autophagy, known to be involved in N remobilization in *Arabidopsis* [53], and/or the presence of CCVs, which have been observed recently at the end of chloroplast dismantling [47]. Another possibility is the degradation of the stromal proteins, such as Rubisco and glutamine synthetase 2, in the senescence-associated vacuoles (SAVs), as observed for tobacco and *Arabidopsis* [46,54]. Among the vacuolar proteases, the cysteine proteases are known to be involved in the senescence processes, and the high contribution of this class of proteases in our experiment confirms the previous studies on oilseed rape [48] and *Arabidopsis* [54,55,56]. Among the cysteine proteases, SAG12 was immunolocalized in SAVs in *Arabidopsis* [54] and was very abundant in senescent leaves of winter oilseed rape in response to low N supply [19]. These data suggest that the protease SAG12 could be involved in the high cysteine protease activities associated with the high N remobilization observed during leaf senescence in response to LN conditions for both genotypes. Conversely, no serine proteases have been identified previously in vacuoles, except a putative subtilisin-like protease in *Arabidopsis* [57]. However, acidic serine protease activity has been observed in SAVs of tobacco (pH 5.2) [46]. Consequently, the enhanced degradation of the soluble proteins of Aviso and Californium under LN conditions (Figure 2b) could be due to an increase in subcellular trafficking involving the acidic cysteine and serine proteases of SAVs, but to our knowledge the accumulation of SAVs in response to restricted N supply has never been investigated.

In addition, a high contribution of aspartic proteases at pH 5 was found in Aviso in response to LN treatment (Figure 4d) and was associated with the identification of a putative vacuolar aspartic protease of *Brassica napus* (GI: 1326165) isolated from zymograms (Figure 5b). This class of proteases presents numerous homologues in the *Brassica napus* genome (Table A1). Additionally, this vacuolar aspartic protease has been previously highlighted on 2-DE gels in response to nitrate limitation in oilseed rape [19] and seems therefore implicated in the efficient leaf proteolysis observed in response to N limitation. However, no aspartic proteases were detected for Californium (Figure 4d and Figure 5b), suggesting that efficient proteolysis can be achieved by the involvement of various sets of proteases.

### 3.2. The High Leaf NRE Observed in Oase, Irrespective of the Level of Nitrate Supply, Is Mainly Related to Efficient Proteolysis Mechanisms at Acidic pH

Irrespective of the nitrate supply, Oase was characterized by a high N remobilization during senescence (Figure 1a,b), the consequences of which were (i) a high amino acid export (Figure 2c,d); (ii) an efficient proteolysis of soluble proteins (Figure 2a,b); and (iii) a high rate of degradation of D1 and lhcb3 proteins compared with other genotypes (Figure 3c,d). These results suggest that the high leaf NRE of Oase is associated with an efficient proteolysis of both stromal and thylakoid-bound proteins. Taken together, the efficient degradation of D1 protein in Oase and the increase in D1 degradation as well as the improvement in N remobilization in response to LN supply for Aviso and Californium all suggest that a high rate of D1 degradation is associated with an efficient N remobilization. To our knowledge, this is the first time that D1 protein degradation has been observed during the time course of senescence in oilseed rape leaves, and it has been clearly associated with a high leaf NRE.

**Figure 8:**
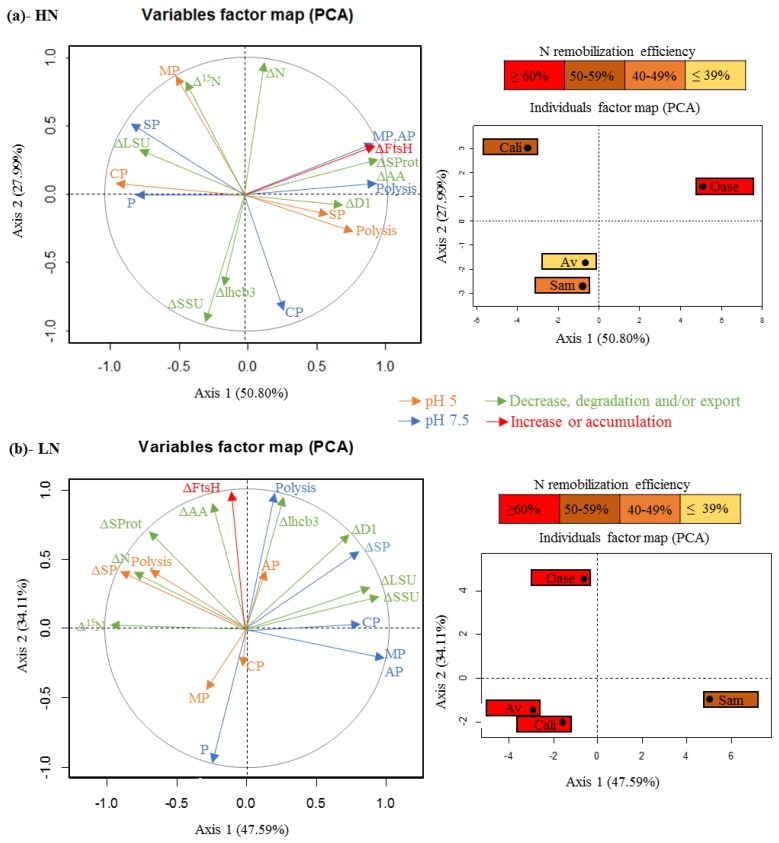
Principal component analysis (PCA) of the criteria of N remobilization determined in response to ample (HN, 3.75 mM; (**a**)) and restricted (LN, 0.375 mM; (**b**)) nitrate applied for 21 days. In both cases the first axis is mainly associated with the total proteolysis at pH 7.5 at D14 and the second axis refers to the N remobilization between D0 and D14. On the individual factor map, the N remobilization in the source leaves is indicated in color for each genotype. Δ^15^N: variation in the amount of ^15^N between D0 and D14; ΔAA: variation in the amount of amino acids between D0 and D14; AP: contribution of aspartic proteases at D14; Av: genotype Aviso; Cali: genotype Californium; CP: contribution of cysteine proteases at D14; ΔD1: variation in the amount of D1 protein between D0 and D14; ΔFtsH: variation in the amount of FtsH between D0 and D14; Δlhcb3: variation in the amount of lhcb3 between D0 and D14; ΔLSU: variation in the amount of the Rubisco large subunit between D0 and D14; MP: contribution of metalloproteases at D14; ΔN: variation in the N amount between D0 and D14; P: contribution of the proteasome at D14; Polysis: total proteolysis activity at D14; Sam: genotype Samouraï; SP: contribution of serine proteases at D14; ΔSProt: variation in the amount of soluble protein between D0 and D14; ΔSSU: variation in the amount of the Rubisco small subunit between D0 and D14.

The degradation of D1 protein in PSII involves two types of chloroplastic proteases, the serine protease, Deg, and the metalloproteases, FtsH [33,36], but the degradation of the chloroplastic proteins is also dependent on the availability of the substrate for proteolysis [37,58,59]. For cyanobacteria, the Deg proteases are known to improve the proteolysis of D1 by FtsH proteases during the degradation of PSII [60]. Under LN conditions, the substantial degradation of the D1 protein for Oase (Figure 3c) is concomitant with an increase of neutral serine proteases (Figure 6d), as shown by principal component analysis (Figure 8), and is correlated with neutral serine protease activity.

Consequently, the preferential degradation of D1 protein observed in Oase in response to N limitation could be related to the contribution of Deg serine proteases, as observed for cyanobacteria [60]. In addition, this could be associated with a higher involvement of CCVs [47]. Indeed, these vesicles are associated with the CV (chloroplast vesiculation) protein, which is believed to destabilize the PSII at the last step of senescence, leading to a greater susceptibility of D1 proteins to degradation by chloroplastic proteases. Consequently, the involvement of CCVs in the high level of D1 protein degradation in the Oase genotype needs to be investigated further.

The efficient proteolysis in both N conditions is linked to a high protease activity at acidic pH, especially the cysteine, aspartic and serine proteases. As suggested below for Aviso and Californium, the significant involvement of these proteases in the proteolysis occurring under LN conditions (Figure 4d) suggests an enhancement of autophagy mechanisms and/or CCVs for the chloroplastic proteins to be sent towards the lytic vacuoles, and/or the occurrence of a higher level of degradation in SAVs.

As reported for Aviso, the substantial contribution of aspartic proteases at pH 5 observed in Oase in response to N limitation (Figure 4d) was associated with an increase in the activity of a putative aspartic protease (GI: 1326165; Figure 5b and Table A1). This aspartic protease was also found on zymograms under HN conditions for Oase (Figure 5a), despite the lack of inhibition of the aspartic proteases (Figure 4c). This can be due to the fact that our inhibitor is not fully active on phytepsins, the family of aspartic proteases reported to be involved in the senescence processes in tobacco [41,61], wheat [62] and *Arabidopsis* [42]. In addition, CND41, the tobacco phytepsin known to be involved in Rubisco degradation, is slightly inhibited by the inhibitor pepstatin A [63]. The aspartic protease detected on zymograms is not similar to CND41 (26% identity) but this protease is close to other phytepsin proteases of *Arabidopsis* (92% identity; Table A1), and, therefore, could be weakly inhibited by pepstatin A. In any case, the high activity of AP in Aviso and Oase under LN conditions is associated with the greatest decrease in soluble protein contents, suggesting a crucial role in proteolysis in response to restricted nitrate supply. In addition, the same aspartic protease (GI: 1326165) was identified in senescing leaves of oilseed rape subjected to nitrate limitation [19] and was observed among the 30 most abundant proteins in dead leaves of oilseed rape [51], making this protease of special interest for understanding the proteolysis associated with leaf N remobilization, especially under restricted N supply.

### 3.3. The Leaf NRE of Samouraï Is Limited between Proteolysis and Amino Acid Export

In both N conditions, the genotype Samouraï is characterized by an efficient degradation of soluble protein (Figure 2a,b), and this is associated with a high involvement of acidic proteases, especially serine and cysteine proteases under LN conditions (Figure 4d and Figure 8). These results agree with the hypothesis of an involvement of autophagy, CCVs and/or SAVs in the mechanisms associated with N remobilization under restricted nitrate supply. In addition, the aspartic proteases are also important under LN conditions (Figure 4d), confirming that they should be investigated to better understand the response to nitrate limitation. However, this high level of proteolysis is not correlated with an efficient leaf N remobilization (Figure 1a,b). The abundance of D1 and lhcb3 proteins remained high in both N conditions throughout the experiment compared with Oase, a genotype characterized by an efficient proteolysis (Figure 3c,d). This low degradation rate could be related to the low amount of FtsHs (Figure 7b), a group of proteases known to be involved in the degradation of the PSII proteins, D1 and LHCII [36,37]. Consequently, the N remobilization of Samouraï could be related to a low level of degradation of the thylakoid-bound proteins of PSII. However, a low level of transmembrane protein degradation could not explain why the degradation of the soluble proteins did not lead to a decrease in the N amount in the source leaf.

Before being transported to the growing organs, the amino acids resulting from proteolysis need to be converted into their transportable forms [15,16] by various enzymes of N metabolism [17]. The fact that no accumulation of the amino acid pool was observed in both N conditions (Figure 2c,d) suggests that leaf N remobilization in Samouraï is not limited by the conversion and/or the export of amino acids, which confirms the previous results [8,18,19]. Therefore, the defect of leaf N remobilization is likely related to a step between proteolysis and amino acid loading into phloem vessels.

Firstly, proteolysis releases a large amount of ammonium [64], which must be detoxified quickly by various enzymes such as glutamine synthetase, glutamate dehydrogenase or asparagine synthase [65]. Consequently, a fault in the detoxification of ammonium in the source leaf of the genotype Samouraï could lead to an ammonium accumulation as a consequence of the high proteolysis observed. These enzymes should be investigated to validate or refute this hypothesis. On the other hand, in both N conditions, a substantial activity of the proteasome system was observed whatever the genotype (Figure 6c,d). The involvement of the proteasome system in leaf senescence was expected and confirms the previous results in *Arabidopsis* [66,67] and oilseed rape [19]. In animal cells, the proteasomes can degrade proteins into peptides, which in turn are degraded into amino acids by a dedicated system [68,69]. A similar pathway has been reported in *Arabidopsis* in response to cadmium toxicity [70], but its occurrence in other conditions (such as mineral limitation) cannot be excluded. Consequently, the low N remobilization of Samouraï, despite an efficient proteolysis and export of amino acids, might be related to a weakness in the degradation and/or the export of oligopeptides that are potentially derived from proteolysis by the proteasome. Detailed study of the export and catabolism of peptides would be required to verify if they are involved in the N remobilization processes. However, in both N conditions in the present study, the degradation of the soluble proteins in Samouraï occurred later compared with other genotypes (significant decrease at D21) (Figure 2a,b). Consequently, export of the products of a late proteolysis may limit N remobilization, with this delay potentially restricting implementation of the mechanisms needed for an efficient N export from the cell before abscission.

## 4. Experimental Section

### 4.1. Experimental Design

Four genotypes of winter oilseed rape (*Brassica napus* L. *cv*. Aviso, Oase, Californium and Samouraï), known to have contrasting foliar N remobilization, were chosen from a preliminary screening of 10 genotypes [8]. Briefly, after germination, plants were transferred to pots (one plant per pot) containing perlite (2 v)/vermiculite (1 v) under greenhouse conditions (thermoperiod of 20 °C (day: 16 h) and 15 °C (night: 8 h)). Plants were supplied with 90 mL of 25% Hoagland nutrient solution (1.25 mM Ca(NO_3_)_2_ 4 H_2_O, 1.25 mM KNO_3_, 0.5 mM MgSO_4_, 0.25 mM KH_2_PO_4_, 0.2 mM EDTA, 2 mM NaFe 3 H_2_O, 14 µM H_3_BO_3_, 5 µM MnSO_4_, 3 µM ZnSO_4_, 0.7 µM (NH_4_)_6_Mo_7_O_24_, 0.7 µM CuSO_4_, 0.1 CoCl_2_) renewed twice a week. From the pot transfer and during the following six weeks the nutrient solution contained 3.75 mM of labeled ^15^N-nitrate (2.5 atom% excess) in order to estimate precisely the leaf N remobilization [71]. After the six weeks of labeling, the ^15^N-nitrate was removed from the nutrient solution and replaced by ^14^N-nitrate (corresponding to Day 0 (D0) of the experiment). The plants were then supplied for 21 days with 25% Hoagland solution (90 mL·day^−1^ per plant) with two different N concentrations: high (HN: 3.75 mM) or low nitrate (LN: 0.375 mM). During the experiment, all leaves were numbered in order of appearance (leaf rank #1 corresponded to the oldest leaf). To study the processes of N remobilization, a leaf mature at D0 undergoing senescence during the experiment, was chosen on the basis of its leaf area (by LI-COR 300 area meter, LI-COR, Lincoln, NE, USA) and chlorophyll content (determined by SPAD: Soil Plant Analysis Development; Minolta, SPAD-502 model) as previously described by Girondé *et al.* [8]. This leaf, called the “source leaf”, presented a mean value for leaf area of 127.91 cm^2^ (±18.60% of variation) and 52.20 (± 5.52% of variation) for SPAD in the four genotypes. This source leaf corresponded to leaf rank number 11 for Californium and leaf rank number 12 for the other genotypes. The source leaf was harvested after 14 (D14) and 21 (D21) days of N treatments. Half of the source leaf was freeze-dried for dry matter quantification and biochemical analysis, and the other half was frozen in liquid nitrogen and stored at −80 °C for further protein quantification and proteolytic assays.

### 4.2. Quantification of Total N and ^15^N Amounts

To determine the foliar N remobilization, the N and ^15^N amounts in the source leaf of the four genotypes were quantified at D0, D14 and D21 by an elemental analyzer (EA3000, EuroVector, Milan, Italy) linked to a continuous flow isotope mass spectrometer (IRMS, IsoPrime GV instruments, Manchester, UK).

### 4.3. Extraction and Quantification of Amino Acids

For the extraction of amino acids, 1 mL of sodium phosphate 100 mM (pH 7.5) was added to 25 mg of freeze-dried leaf samples and incubated twice (30 min, 80 °C) with 1 mL of 80% and 50% ethanol, respectively. After each incubation the resulting mixtures were centrifuged (10 min, 12,000 *g*, 4 °C) and the supernatants containing amino acids were pooled, evaporated and re-suspended in water. To determine the amino acid concentration, 1 mL of ninhydrin reagent (112 mM ninhydrin and 3.85 mM tin chloride in 100 mM citrate buffer pH 5, 50% Dimethylsulfoxide (DMSO), *v*/*v*) was added to 100 µL of amino acid extract (adapted from [72]). After incubation for 20 min in boiling water and 10 min on ice, 5 mL of 50% ethanol were added and the absorbance was read at 570 nm. The concentration of amino acids was calculated using a range of l-leucine standards from 0.2 to 2 mM.

### 4.4. Extraction and Quantification of Soluble Proteins

The soluble proteins of the source leaf were extracted from 200 mg of fresh matter previously crushed in a mortar with liquid nitrogen in the presence of 500 µL of citrate (20 mM)-phosphate (160 mM) buffer (pH 6.8) containing 50 mg of polyvinylpolypyrrolidone (PVPP). The homogenate was centrifuged for 1 h at 12,000 *g* (4 °C) and the supernatant, containing the soluble proteins, was adjusted in a microtube to 500 µL with citrate-phosphate buffer. The concentration of the soluble proteins was quantified by protein-dye staining [73] using bovine serum albumin (BSA) as standard.

### 4.5. Determination of Proteolytic Activities Using the Rubisco Large Subunit as Substrate

In order to identify the proteases involved in the proteolysis processes associated with leaf N remobilization in the four genotypes, a test of proteolytic activities using the large subunit (LSU) of Rubisco as a substrate was performed in the presence of specific inhibitors of the different classes of proteases at pH 5 and 7.5 as set out in our earlier paper [74]. Because this previous study on senescing leaves of oilseed rape demonstrated that there were no significant differences between biological repetitions for measurements of soluble protein content and the proteolytic activity test, the three biological replicates of the soluble protein extract (PE) obtained from source leaf at D14 were pooled for the proteolytic activities. The pool of protein extracts was either incubated at 37 °C (*t*_0_*+*Δ*t*) or not (*t*_0_) at pH 5 in sodium acetate buffer (125 mM final concentration containing 0.8% β-mercaptoethanol, *v*/*v*) for proteolytic activities at acidic pH (proteases in vacuole and apoplast) or at pH 7.5 in Tris-HCl buffer (125 mM final concentration containing 0.8% β-mercaptoethanol, *v*/*v*) for proteolytic activities at neutral pH (proteases in stroma and cytosol). Iodoacetamide (14.5 mM) was used as an inhibitor of cysteine proteases (PE + CPI) and aprotinin (68 µM) as a serine protease inhibitor (PE + SPI). For characterization of metalloprotease and aspartate protease activities, 11 mM of 1–10 phenanthroline (PE + Me + MPI) and 20.6 µM of pepstatin A (PE + Me + API) were used, respectively. For the inhibition of the proteasome system, 20 µM of MG132 (benzyloxycarbonyl-leu-leu-leucinol) were used (PE + DMSO + PI) but, due to the localization of proteasome in the nucleus and cytosol, the inhibition was only performed at pH 7.5. Due to the preparation of 1–10 phenanthroline (MPI) and pepstatin A (API) in methanol, and the preparation of MG132 in dimethylsulfoxide (DMSO), the total protease activity was also carried out with 0.5% methanol (*v*/*v*; PE + Me) and 0.5% DMSO (*v*/*v*; PE + DMSO) as control.

The conditions of the proteolysis tests had to be carefully adapted to the N treatments, the genotype and the pH of incubation. Consequently, the amount of proteins used for these tests differed between pH conditions: a total of 10 µg (3.3 µg of each triplicate) was used for the proteolytic activities at pH 5, and a total of 6 µg (2 µg of each triplicate) for the proteolytic activities at pH 7.5. In the same way, the duration of incubation was adapted at pH 5 as follows: 45 min for the HN treatment except for *cv.* Californium, which needed 55 min; 20 min for the LN treatment except for Californium, which required 30 min. At pH 7.5, the duration of incubation was 60 min for Oase and 90 min for the other genotypes in both N conditions.

At *t*_0_, or after the incubation at 37 °C (*t*_0_*+*Δ*t*), 2X Laemmli buffer [75] containing β-mercaptoethanol (5%; *v*/*v*) was added to the protein samples (one volume of 2X Laemmli buffer per volume of protein extract) before protein denaturation for 7 min in boiling water. The resulting samples were loaded on Stain Free SDS-PAGE (Mini-PROTEAN TGX Stain Free, 4%–15% acrylamide, Bio-Rad^®^). The abundance of the Rubisco large subunit (LSU) at *t*_0_ and after the incubation at 37 °C (*t*_0_*+*Δ*t*) was determined by quantification of the volume of the LSU band (V_LSU_) using Image Lab software (Bio-Rad^®^, Marnes La Coquette, France). To compare the genotypes, the proteolytic activities (% of degradation of LSU: %Deg) were defined for 20 min at pH 5 and 60 min at pH 7.5, by the following equation:

%Deg = [[(V_LSU_*t*_0_ − V_LSU_*t*_0_*+*Δ*t*)*/*T] × T_x_] × 100/V_LSU_*t*_0_ (1)

where V_LSU_*t*_0_ and V_LSU_ *t*_0_*+*Δ*t* correspond to the peak volume of LSU band at *t*_0_ and *t*_0_*+*Δ*t*, respectively, T refers to the time of incubation and T_x_ is the time of incubation used to compare genotypes (20 min at pH 5 or 60 min at pH 7.5).

The inhibition of LSU proteolysis (%Inh) by the different protease inhibitors was determined as follows:

%Inh = (%Deg control − %Deg inh)/(%Deg control/100)

where %Deg control and %Deg inh are the % of degradation of LSU calculated by using Equation (1) in the control sample (without protease inhibitor) and in the sample in presence of a specific protease inhibitor, respectively. Previous results [74] have demonstrated that a significant difference in LSU degradation is observed when the rate of degradation between HN and LN treatments is at least doubled. Based on these data, this threshold value (*i.e.*, a two-fold factor) was used to consider that the rate of degradation is strongly affected by LN treatment for a given genotype or to compare the effect of the inhibitors of proteases.

### 4.6. Detection of Proteolytic Activities by Zymograms

*In gelo* protease activities were performed as reported by Dominguez and Cejudo [76]. For zymograms, the three biological replicates of soluble protein extracts at D14 were pooled and a total of 75 µg were loaded per lane. After adding 2X Laemmli buffer without β-mercaptoethanol [75], the proteins were separated by SDS-PAGE using a 5.5% polyacrylamide (*w*/*v*) stacking gel and a 10% polyacrylamide (*w*/*v*) resolving gel co-polymerized with gelatin (0.1%, *w*/*v*). The resulting gels were incubated for 45 min in 25 mL 2-propanol 25% (*v*/*v*) and rinsed with water. After 16 hours of incubation (30 °C) in 100 mL of 100 mM sodium acetate buffer at pH 5 containing 10 mM dithiothreitol (DTT), the gels were stained with 0.25% Coomassie R-250 blue in 50% methanol and 10% acetic acid (*v*/*v*). The proteolytic activities were revealed as clear band using a destaining solution (20% methanol, 10% acetic acid, *v*/*v*) and the gels were analyzed using Image Lab software (Bio-Rad^®^).

Bands showing proteolytic activity on the zymograms were excised and washed several times with water. After drying, a trypsin digestion was performed overnight with a dedicated system (MultiPROBE II, Perkin-Elmer, Waltham, MA, USA). The resulting peptides were extracted from the gel by two incubation periods of 15 min in a 0.1% CH_3_CN solution (*w*/*v*). Peptides were then dried and dissolved in starting buffer (3% CH_3_CN and 0.1% HCOOH, *w*/*v*) for chromatographic elution. Peptides were enriched and separated using lab-on-a-chip technology (Agilent, Massy, France) and fragmented using an on-line XCT mass spectrometer (Agilent). The ESI LC-MS/MSdata were converted into DTA-format files that were further searched for protein identification with MASCOT Daemon (Matrix Science, [77]) in the NCBInr-protein sequence database, Viridiplantae (green plants), and in the Brassica EST database (Brassica Genome Gateway 2007, [78]). The spectra of each peptide were checked manually.

### 4.7. Immunodetection

Western blots of the large (LSU) and small subunit (SSU) of Rubisco were performed using the soluble protein extract described below (2.4). For the immunodetection of insoluble proteins (D1 and lhcb3) that belong to photosystem II (PSII) and the filamentation temperature-sensitive H proteases (FtsHs), a specific method of protein extraction was performed [79]: 200 mg of frozen fresh matter were crushed in the presence of liquid nitrogen and 50 mg of PVPP and were re-suspended in 1.75 mL of acetone containing 10% trichloroacetic (*v*/*v*). The extract was centrifuged at 16,000 *g* (3 min, 4 °C) and the pellet was rinsed as described in Desclos *et al.* [79]. The resulting pellet was resuspended in R2D2 buffer (5 M urea, 2 M thiourea, 2% CHAPS, 2% *N*-decyl-*N*,*N*-dimethyl-3-ammonio-1-propanesulfonate, 20 mM dithiothreitol, 5 mM Tris(2-carboxy-ethyl)phosphine, 0.5% IPG buffer pH 4 to 7 (GE Healthcare)) [80]. The concentration of the protein extract was quantified by protein-dye staining [72] as described previously for soluble proteins (2.4).

For the Western blot, the three biological replicates of protein extracts were pooled and 15 µg were loaded for protein immunodetection. This amount of protein was determined in order to have the best immunodetection for all the studied proteins. After denaturation with Laemmli 2X buffer [75] containing 5% β-mercaptoethanol (*v*/*v*), the proteins were separated by SDS-PAGE (5.5% and 10% polyacrylamide (*w*/*v*) for the stacking and the resolving gel, respectively) and transferred to a polyvinylidene difluoride (PVDF) membrane as described by Desclos *et al.* [79]. The immunodetection was made using specific polyclonal antibodies from rabbit provided by Agrisera^®^ (Vannas, Sweden) for the LSU (1/10,000) and the SSU of Rubisco (1/5000), the lhcb3 (1/2000), the D1 proteins (1/20,000) and the FtsH proteases (1/1000). The primary antibodies were detected by secondary antibodies from goat coupled with alkaline phosphatase (1/12,000, Bio-Rad^®^) for LSU, SSU, lhcb3 and FtsH. For D1 protein, secondary antibodies from goat coupled with peroxidase were used (1/10,000, Bio-Rad^®^) and detected by enhanced chemiluminescence using a ProXPRESS 2D proteomic Imaging System (PerkinElmer, Courtaboeuf, France). Primary and secondary antibodies were diluted in Tris buffer saline, Tween 20 (Tris 10 mM, NaCl 150 mM, pH8, Tween 20 0.15%, *v*/*v*) containing 5% milk (*v*/*v*) to avoid non-specific hybridization.

### 4.8. Statistical Analysis

The normality of the data was examined with the Ryan-Joiner test at 95%. Analysis of variance (ANOVA) and the Tukey test were used to compare the means. When the normality law of data was not respected, the non-parametric test of Kruskal-Wallis was carried out. Statistical significance was postulated at *p* < 0.05. The source of variation and the correlations were determined by an ANOVA and the Pearson test, respectively (* p < 0.05; ** p < 0.01; *** *p* < 0.001). Three biological repetitions were used (*n* = 3) and all the data presented are expressed as the mean ± standard error (SE), when the triplicates were not pooled.

## 5. Conclusions

The present study confirms that proteolysis is a key factor to improve N remobilization in leaves of oilseed rape [8,20] and that a high N remobilization in leaves at vegetative stages is related to an efficient degradation of both soluble and thylakoid-bound proteins of the chloroplasts. A strong correlation between the leaf NRE and the rate of D1 degradation was observed in both nitrate conditions. Further studies are needed to determine if the rate of D1 degradation can be used as a protein indicator to identify genotypes with high leaf NRE and especially with reduced N loss in fallen leaves. The efficient degradation of the D1 protein seems to be associated with acidic proteolytic activity and serine proteases at neutral pH, especially for genotype Oase under low N supply. As for cyanobacteria, the serine protease Deg could be involved in the improvement in D1 protein degradation.

Efficient proteolysis of the soluble proteins under restricted N supply was associated with proteolytic activity at acidic pH, especially the activities of serine, cysteine and aspartic proteases. These results suggest an important subcellular trafficking of the chloroplastic proteins to the vacuoles, including autophagy mechanisms and/or CCVs. In addition, due to the localization of serine protease activities in SAVs in *Arabidopsis* leaves, further investigation is required to identify SAVs in oilseed rape leaves and to characterize their role in N remobilization in response to restricted N supply. Future development based on biochemical fractionation and purification of organelles (vacuole, chloroplasts, *etc.*) or immunodetection of proteases at subcellular level will be necessary to confirm the relative contributions of the different classes of proteases. Finally, the acidic aspartic protease GI: 1326165, which was identified in the present study during the leaf senescence of two genotypes and in two other studies in response to a nitrate limitation for the genotype Capitol, should be characterized to understand its role in proteolysis during the senescence triggered by N limitation.

## Appendix

**Table A1:** Score and number of peptides found during identification of proteases from zymograms and their putative homologue in the *Brassica napus* and *Arabidopsis* genomes. The bands corresponding to proteolytic activities at pH 5 on the zymograms were analyzed by LC-MS/MS to identify the proteases responsible for the proteolytic activity. The proteases identified with success are presented with their accession number, score and the number of peptides identified. The NCBI BLAST database was used to identify proteases in the *Brassica napus *and *Arabidopsis thaliana *genomes.

Genotype/N Treatment	Score/Number of Peptides	Proteases Name (Organisms)/NCBI Accession No	Result of BLAST Protein-Protein (*Brassica napus*)/NCBI Accession No./% Identity	Result of BLAST Protein-Protein (*Arabidopsis thaliana*)/NCBI Accession No./% Identity
Aviso/LN	49/7	Aspartic proteases (*Brassica napus*)/gi|1326165	BnaA09q47450D/gi|674898012/98%	AT1G11910/gi|222424506/95%
			BnaC08g15160D/gi|674901714/93%	Aspartic proteinase/gi|1354272/93%
			BnaA08g25040D/gi|674912117/92%	Aspartic proteinase A1/gi|15221141/92%
Oase/HN	22/3		BnaC04g19410D/gi|674924641/79%	Aspartic proteinase A2/gi|22330379/80%
			BnaA03g59920D/gi|674908220/78%	Aspartic proteinase A3/gi|15233518/66%
Oase/LN	132/12		BnaA09g20460D/gi|674916523/67%	
			BnaC09g22820D/gi|674938912/67%	
